# Efficient and Accurate Brain Tumor Classification Using Hybrid MobileNetV2–Support Vector Machine for Magnetic Resonance Imaging Diagnostics in Neoplasms

**DOI:** 10.3390/brainsci14121178

**Published:** 2024-11-25

**Authors:** Mohammed Jajere Adamu, Halima Bello Kawuwa, Li Qiang, Charles Okanda Nyatega, Ayesha Younis, Muhammad Fahad, Salisu Samaila Dauya

**Affiliations:** 1 Department of Electronic Science and Technology, School of Microelectronics, Tianjin University, Tianjin 300072, China; liqiang@tju.edu.cn (L.Q.); ncharlz@tju.edu.cn (C.O.N.); ayesha@tju.edu.cn (A.Y.); 2 Department of Computer Science, Yobe State University, Damaturu 600213, Nigeria; ssdauya@ysu.edu.ng; 3 Center for Distance and Online Education, Lovely Professional University, Phagwara 144411, India; 4 Department of Biomedical Engineering, School of Precision Instruments and Opto-Electronics Engineering, Tianjin University, Tianjin 300072, China; halima@tju.edu.cn; 5 Department of Electronics and Telecommunication Engineering, Mbeya University of Science and Technology, Mbeya P.O. Box 131, Tanzania; 6 School of Electrical and Information Engineering, Tianjin University, Tianjin 300072, China; mfahadgull77@tju.edu.cn

**Keywords:** MR images, brain tumor, classification, machine and deep learning, MobileNetV2, SVM

## Abstract

Background/Objectives: Magnetic Resonance Imaging (MRI) plays a vital role in brain tumor diagnosis by providing clear visualization of soft tissues without the use of ionizing radiation. Given the increasing incidence of brain tumors, there is an urgent need for reliable diagnostic tools, as misdiagnoses can lead to harmful treatment decisions and poor outcomes. While machine learning has significantly advanced medical diagnostics, achieving both high accuracy and computational efficiency remains a critical challenge. Methods: This study proposes a hybrid model that integrates MobileNetV2 for feature extraction with a Support Vector Machine (SVM) classifier for the classification of brain tumors. The model was trained and validated using the Kaggle MRI brain tumor dataset, which includes 7023 images categorized into four types: glioma, meningioma, pituitary tumor, and no tumor. MobileNetV2’s efficient architecture was leveraged for feature extraction, and SVM was used to enhance classification accuracy. Results: The proposed hybrid model showed excellent results, achieving Area Under the Curve (AUC) scores of 0.99 for glioma, 0.97 for meningioma, and 1.0 for both pituitary tumors and the no tumor class. These findings highlight that the MobileNetV2-SVM hybrid not only improves classification accuracy but also reduces computational overhead, making it suitable for broader clinical use. Conclusions: The MobileNetV2-SVM hybrid model demonstrates substantial potential for enhancing brain tumor diagnostics by offering a balance of precision and computational efficiency. Its ability to maintain high accuracy while operating efficiently could lead to better outcomes in medical practice, particularly in resource limited settings.

## 1. Introduction

Brain tumors are abnormal cell proliferations that severely impair neurological functions by compressing or infiltrating healthy tissue. They can be classified as benign or malignant, with malignant tumors posing greater risks due to aggressive growth and potential metastasis, as categorized by the World Health Organization’s 2021 CNS tumor classification into grades I to IV based on aggressiveness, notably including malignant gliomas like glioblastomas (WHO grade IV) [1], which are among the most lethal, leading to rapid neurological deterioration, cognitive deficits, and significantly reduced survival rates. In contrast, grades I and II are considered low-grade tumors, while grades III and IV exhibit rapid growth and the ability to infiltrate adjacent healthy cells. The classification of brain tumors using Magnetic Resonance Imaging (MRI) is essential, as it allows radiologists and clinicians to accurately determine tumor type, assess its extent, and provide appropriate treatment options [2,3].

Beyond the structural impacts, brain tumors can disrupt neural pathways and neurotransmitter systems. The abnormal growth of tumor cells can significantly affect the networks of neurons that underlie cognitive and motor functions. Depending on the tumor’s location, critical functions such as movement, speech, memory, and emotional regulation can be impaired. For example, tumors in the frontal lobe may affect executive functions, while those in the occipital lobe could lead to visual disturbances [4,5].

Brain tumors can disrupt neurotransmitter systems by exerting pressure on specific brain regions, altering synaptic transmission and neural communication. This can lead to motor deficits, cognitive impairments, and emotional regulation changes, with gliomas often linked to imbalances in glutamate signaling, exacerbating neural excitotoxicity and damaging healthy tissue [6,7].

Brain tumors are classified by location, texture, form, and size, with meningiomas, pituitary tumors, and gliomas [8,9,10] being the most prevalent types. In 2020, the World Cancer Research Fund International reported 18.1 million new cancer cases globally, with brain cancer being the 19th most common type, while Pakistan’s Global Cancer Observatory noted 4770 new brain cancer cases, ranking it 11th among all malignancies [11,12]. Traditional methods for diagnosing malignant tumors often involve invasive surgical procedures to extract tissue samples for analysis, leading to a need for less intrusive and more effective techniques.

In terms of newly registered and fatal recorded cases, brain tumors ranked 11th and 9th, respectively, among all malignancies, with 4770 new diagnoses and 3934 resulting in death [13,14]. Malignant tumor diagnosis typically involves the surgical extraction of a tissue sample for further testing, allowing for the classification of the tumor and recommendation of appropriate treatments. Traditional diagnostic methods were often invasive and complex, leading to the development of more effective and precise techniques for categorizing brain scans and identifying tumors. 

Magnetic Resonance Imaging (MRI) is the standard for brain tumor detection, but variability in tumor characteristics complicates early diagnosis, prompting the need for advanced methods; this study proposes a hybrid model combining MobileNetV2 with a Support Vector Machine (SVM) to enhance tumor classification and diagnostic performance through machine learning techniques [15,16]. The MobileNetV2 model optimized by the Contracted Fox Optimization Algorithm [17] and EfficientNet models for brain tumor classification [18] were used.

These non-invasive methods utilize various imaging techniques to visualize brain tumors, such as Magnetic Resonance Imaging (MRI), Computer Tomography (CT) scans, Single-Photon Emission Computerized Tomography (SPECT), X-rays, and Positron Emission Tomography (PET) [19]. MRI scans are widely acknowledged as a fundamental necessity for medical practice in clinics [20,21]. The images produced by Magnetic Resonance Imaging (MRI) are more distinct and precise compared to Computerized Tomography (CT) scans. For example, when examining soft tissues like brain tumors, MRI images are preferred over CT scans or X-rays [22].

Distinguishing between normal and malignant brain tissues is crucial for tumor identification, yet detecting brain tumors is challenging due to variations in location, size, and shape. Processing medical images through segmentation, detection, and classification is essential for early identification, as brain tumors can be persistent and deadly. Manual examination of MRI images by healthcare professionals may lead to human error, misdiagnosis, and inefficiencies.

Given the low likelihood of early tumor detection, an automated system was developed to categorize, segment, and detect brain tumors, addressing the limitations of manual techniques through MR image preprocessing, feature extraction, and classification using a supervised learning algorithm [23]. The crucial stage in this procedure is categorization, carried out by either machine learning or deep learning.

An expert system utilizing machine learning can perform tasks such as image categorization in the medical field for both therapeutic and educational purposes, with preprocessing being crucial for feature extraction. Various powerful machine learning algorithms, such as ANN, BPNN, SVM, K-NN, and PNN, are employed to identify and categorize brain tumors from different datasets [24]. Deep learning (DL) is a rapidly growing technique that is becoming increasingly popular and widely studied in several domains, particularly in the analysis of medical imaging [25]. Deep learning enhances adaptability and proficiency by processing non-uniform input across multiple levels. Feature extraction can be placed at each subsequent layer and then sent [26]. The primary characteristic of deep learning is the automatic acquisition of relevant data from a dataset. The system automatically identifies and categorizes images based on their main features [27]. 

We chose to use MobileNetV2 in our research because of its lightweight architecture, which is highly efficient for deployment on mobile and edge devices. Furthermore, the selection of SVM is based on its resilience in handling non-linear data and its shown capability to attain exceptional accuracy in classification tasks. The main contribution of our research is developing a hybrid model that combines the feature extraction capabilities of MobileNetV2 with the classification performance of SVM. This combination results in improved precision and computational effectiveness in identifying brain cancers. The key contributions of our research include:

(a) Creating a strong hybrid model that combines MobileNetV2 and SVM.
(b) Exhibiting enhanced precision in categorization and increased computational speed.
(c) Performing thorough assessments on the Kaggle MR images dataset to verify the model’s efficacy.

## 2. Literature Review

This literature discusses the contributions of several researchers who have dedicated their efforts to using new techniques for identifying brain tumors and reaching encouraging results.

Various CNN-based architectures, such as AlexNet, VGGNet, and ResNet, have been widely used for brain tumor classification due to their ability to capture detailed spatial features from MRI images. VGG16 and ResNet have shown high accuracy but at the cost of significant computational resources, making them less practical for real-time applications [28]. ResNet-based models, such as ResNet-18 with SVM, have improved classification performance by addressing the vanishing gradient problem, though they still suffer from high computational complexity [29].

Hybrid approaches combining CNNs with machine learning classifiers, such as SVM, have been proposed to enhance performance. For instance, Senan et al. introduced a ResNet-18 + SVM model, achieving an accuracy of 95.1%, but the model’s complexity remains a challenge in resource-constrained settings [30,31]. In contrast, the proposed MobileNetV2–SVM hybrid model utilizes MobileNetV2’s depth-wise separable convolutions, significantly reducing computational cost while maintaining high accuracy. Compared to models like VGG16 and ResNet, this hybrid model is more efficient, making it suitable for real-time applications in environments with limited resources, such as mobile devices or edge computing platforms.

The researcher developed a unique strategy for categorizing brain tumor MRI images by integrating fuzzy and brainstorming optimization methods [32]. The process of fuzzy optimization involved multiple iterations to identify the optimal network topology. Additionally, brainstorming optimization was given particular importance and focused on determining cluster centers [33], diseases [34,35,36,37], and migraines [38]. The researchers conducted experiments using the BraTS 2018 dataset and obtained impressive results, including an accuracy of 93.85%, sensitivity of 95.77%, F1 score of 95.42%, and precision of 94.77%.

In previous research, the author utilized the modulo and hyper-column approach to develop a network known as BrainMRNet [39]. The raw images underwent processing before the application of the attention module, which exerted control over the convolutional layer and the important regions of the image. The hyper-column approach was extensively utilized in the convolutional layers of the BrainMRNet model. By utilizing the data from each layer to construct the array tree of the final layer, the accuracy of this strategy was determined to be 96.05%.

A researcher proposed a novel approach for segmenting and categorizing brain tumors by employing active deep learning-based feature selection. A saliency map was generated through contrast enhancement and subsequent thresholding to convert it into a binary representation. Furthermore, the InceptionV3 pre-trained model was employed to extract deep features. These features were then combined with the dominating rotated LBP features to enhance the accuracy of the texture analysis [40].

Next, this study employed the SoftMax function to arrange the concatenated vectors, applying particle swarm optimization to obtain their optimal value (PSO). This study employed the BraTS 2017 and BraTS 2018 datasets. The risk scores for the core, total, and enhanced tumors on the BraTS 2017 dataset were 83.73%, 93.7%, and 79.95%, respectively. The BraTS 2018 dataset yielded 88.34%, 91.2%, and 81.8% accuracy rates. T1C, T1, Flair, and T2 are crucial MRI image sequences for detecting brain cancer. Ref. [41] developed a method to integrate their textural and structural features. This research employed a Daubechies wavelet kernel and discrete wavelet transforms to achieve its objective.

Subsequently, a partial differential diffusion filter was utilized to remove any artifacts effectively. Next, a global thresholding technique was used to divide the areas of the lesions. An analysis of five distinct BraTS datasets found that the results obtained by combining the images were better than those obtained by using each sequence individually. This supports the effectiveness of the proposed approach. The approach demonstrates an accuracy rate of 87%, a sensitivity of 92%, and a specificity of 80%.

In Ref. [42], a method that combines CNN’s [43] capability to distinguish between cancerous and non-cancerous tumors with a publicly accessible dataset named BraTS 2015 was developed. This method was used to identify MRI images of brain tumors. Deep learning approaches have gained popularity in recent years for image categorization due to their superior performance.

To detect brain tumors in MRI data, Ref. [44] proposed a hybrid ensemble technique based on the Majority Voting Method. This approach utilizes Decision Tree (DT) and K-nearest neighbor (KNN) algorithms. The classification [45] task utilized KNN–RF–DT, a hybrid ensemble classifier incorporating the Majority Voting technique.

Feature extraction was performed using SWT, PCA, and GLCM, while segmentation was achieved using Otsu’s threshold technique. The method employed conventional classifiers to enhance performance. These classifiers have the advantage of requiring small datasets and having minimal processing time complexity, making them ideal for use by individuals with less knowledge. The approach achieved a 97.305% accuracy when evaluated on a dataset consisting of 2556 images. The training set accounted for 85% of the dataset, while the remaining 15% was used for testing. In Ref. [46], a differential deep convolutional neural network (CNN) [47] was used to detect and categorize different types of brain tumors [43,48] using MRI data. Classifying brain tumors using MRI was a difficult task due to various factors such as the intricate nature of the brain, the overlapping of tissues, and the great density of the brain. The suggested model uses differential deep-CNN operators to extract additional differential feature maps from the initial CNN feature maps. This enhances the performance of the technique. The model achieved a 99.25% accuracy rate after being validated and trained on a dataset of 25,000 MRI brain images, which included both diseased and normal cases.

The researchers in Ref. [49] successfully detected brain tumors using the U-NET CNN and fuzzy logic architecture. The U-NET architecture was employed in this approach, incorporating CNN classification, fuzzy logic-based edge detection, and contrast enhancement techniques [50]. Before applying a dual tree-complex wavelet transform (DTCWT) at many scales on images from various sources, contrast enhancement was performed. The modified images were analyzed using a fuzzy logic-based edge detection (FLBED) approach to identify the edges. The brain’s decomposed sub-band images were classed using the U-NET CNN classification algorithm, resulting in features that can be used to differentiate meningioma brain tumors [51,52] from others. The accuracy of this approach was 98.59% when compared to various recently developed algorithms. Ref. [53] introduced a hybrid deep learning model designed to classify gliomas [37,54,55,56], meningiomas, and pituitary tumors. GoogleNet was used as the basis for a basic convolutional neural network (CNN) structure that was employed to create the model. In order to enhance the model’s expressiveness, this study excluded the last five layers of GooleNet and introduced an additional 15 layers. This feature map also utilized the leaky ReLU activation function. The proposed model surpassed all other employed methods, achieving an accuracy of 99.67%, precision of 99.6%, F1 score of 99.66%, and recall of 100%.

M. Senan et al. [57] highlighted the need for early and precise medical diagnosis of brain cancer. They also underlined the effectiveness of computer-aided diagnostic tools in aiding clinicians in making correct diagnoses, particularly in the field of machine and deep learning. Using ResNet-18, AlexNet, and SVM, multiple studies employ a combination of deep learning and machine learning to categorize and diagnose brain tumors. After applying an average filter to enhance MRI images, deep learning techniques were utilized to extract dependable and important key features using deep convolutional layers. The MRI dataset consists of 3060 images representing three unique cancer [58] forms and one normal tissue type. The AlexNet + SVM hybrid technique demonstrated superior performance compared to previous methods, achieving an accuracy of 95.10%, specificity of 98.50%, and sensitivity of 95.25%.

According to Ref. [59], over 4000 CT individuals from the SCAPIS and IGT cohort were used to train and assess four convolutional neural network architectures: ResUNET, UNET++, Ghost-UNET, and Ghost-UNET++ for segmentation techniques for the liver, spleen, skeletal muscle, bone marrow, cortical bone, and fat tissue deposits.

According to Ref. [60], brain tumors were a prominent cause of mortality. The authors also emphasized the importance of early detection for effective treatment. While a biopsy was required for the usual categorization of brain tumors, it was not consistently performed before surgery. Thanks to advancements in machine learning and other technologies, radiologists now have the ability to detect malignancies using MRI images, eliminating the need for intrusive therapies. A CNN-based architecture, which is a novel hybrid technique, was proposed for classifying the MRI images of three separate brain tumors.

The brain MRI scans identified a total of 1426 gliomas, 708 meningiomas, and 930 pituitary tumors. Additionally, 396 scans of healthy brain tissue were utilized to evaluate the method’s effectiveness. A Support Vector Machine (SVM) classifier utilizing GoogleNet as a feature extractor achieved an accuracy rate of 98.1%. Sarmad Maqsood et al. [12] proposed a precise method for radiologists to identify and categorize brain tumors accurately. The researchers first employed linear contrast stretching to enhance the clarity of the edges in the original image. Next, they employed a deep neural network architecture of 17 layers specifically designed to segment brain tumors. Explainable Artificial Intelligence (XAI) was employed in this instance, and the results indicated that the proposed method surpassed current techniques and had the potential to enhance the field of medical imaging. Surgeons, as well as physicians, possess greater awareness regarding the anatomical structure of individuals’ organs [61,62]. Implementing the SR model in endoscopic imaging has the capacity to transform medical diagnosis along with an operation, greatly enhancing the accuracy and efficiency of endoscope treatments [63].

While several previous studies have achieved high accuracy rates using various machine learning and deep learning techniques, the proposed hybrid model (MobileNetV2 + SVM) offers distinct advantages. For instance, compared to the approach by Senan et al., which achieved an accuracy of 95.1% using the ResNet-18 + SVM hybrid model [57], our model not only improves accuracy but also enhances computational efficiency due to MobileNetV2’s lightweight architecture. This makes it more suitable for deployment in resource-constrained environments, such as mobile or edge devices. Additionally, our model demonstrates superior performance in terms of AUC scores (glioma: 0.99, meningioma: 0.97) compared to traditional methods like BrainMRNet [39], which achieved an accuracy of 96.05% but is lacking in terms of resource efficiency.

However, it is important to note that while our hybrid approach excels in classification performance and computational speed, the complexity added by combining MobileNetV2 with SVM could be a limitation in certain clinical environments where rapid real-time diagnosis is necessary. Methods like U-Net combined with CNN, as used by Maqsood et al. [12], offer simpler implementations with slightly lower computational overhead but may sacrifice classification precision in specific tumor classes.

### Research Gap

Although significant advancements have been achieved in the categorization of brain tumors using deep learning and machine learning techniques, numerous crucial deficiencies still remain. Existing techniques frequently face challenges in achieving harmony between computing efficacy and superior classification precision. Several methods require significant processing resources, making them unsuitable for real-time applications in environments with limited resources. In addition, there is a significant lack of powerful methods that successfully integrate deep learning with conventional machine learning classifiers to enhance performance. Table 1 presents the summary of the existing literature in this field. Our research tackles these difficulties by introducing a hybrid model that combines the lightweight design of MobileNetV2 for fast feature extraction with the strong classification capabilities of SVM.

## 3. Proposed Methodology

The proposed model integrates the MobileNetV2 architecture with an SVM classifier to improve classification accuracy. MobileNetV2’s depth-wise separable convolutions efficiently extract features from MR images, and we modified the architecture by adding bottleneck layers to enhance feature refinement. Instead of using a fully connected layer for classification, we replaced it with an SVM classifier, which excels in handling complex and non-linear data.

The integration happens at the global average pooling layer, where extracted features from MobileNetV2 are passed to the SVM for final classification. This hybrid approach combines MobileNetV2’s strong feature extraction capabilities with SVM’s robustness in distinguishing between tumor types, improving the model’s performance while reducing overfitting.

### 3.1. Proposed Approach Architecture

**Figure 1:**
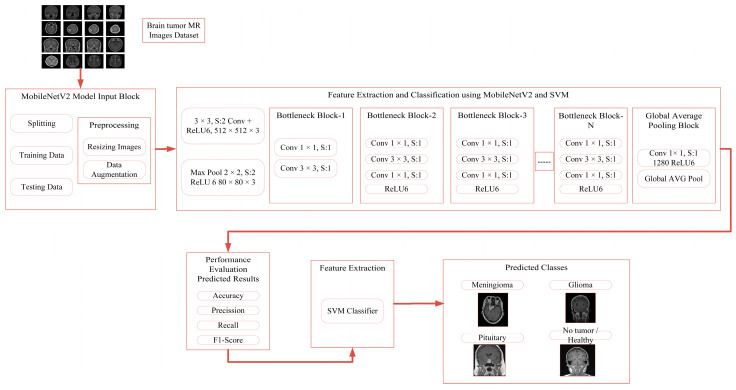
Neural network architecture of the proposed approach.

Figure 1 provides a pictorial representation of the proposed neural network architecture. The MobileNetV2 architecture uses an input image with dimensions of 512 × 512 pixels and three color channels, namely red, green, and blue. The initial convolutional layer uses 3 × 3 filters to identify fundamental characteristics, while the Rectified Linear Unit (ReLU) activation function introduces non-linearity, enabling the model to recognize intricate patterns. A max pooling layer reduces the spatial dimensions of feature maps, enhancing feature recognition regardless of size or orientation. 

Bottleneck blocks are recurring blocks of layers used to process characteristics further, consisting of convolutional layers of varying dimensions. The number of blocks varies based on the network’s specific configuration. Global average pooling is applied before the final classification step to reduce each feature map to one value, reducing the number of model parameters and the risk of overfitting.

After global average pooling, an activation function of ReLU and a fully connected layer with 1280 units are used to summarize the information and process it for the final classification. The architecture uses a Support Vector Machine (SVM) classifier instead of a normal classifier for classification tasks, which can handle non-linear associations and handle the complexity and uncertainty of image data. The SVM classifier is known for its robustness towards various classification methods.

Integrating MobileNetV2 with a Support Vector Machine (SVM) combines deep learning and machine learning for brain tumor classification. MobileNetV2 efficiently extracts high-level features from MRI images, while SVM excels in handling complex, high-dimensional data and creating optimal decision boundaries, especially in imbalanced datasets. This hybrid approach addresses the limitations of traditional CNNs with SoftMax classifiers by improving the model’s ability to handle non-linear class separability and subtle variations in medical data. As a result, the MobileNetV2–SVM model enhances classification accuracy and robustness compared to standard deep learning methods. Convolutional Layers:

(1) $$\left(i\times k\right)\left(x,y\right)=\sum_{i=0}^{k-1}\;\sum_{j=0}^{k-1}\;I\left(x+i,y+j\right).K\left(i,j\right)\;$$

In Equation (1), *I* represents the input image, *K* is used for the kernel (filter), *x* and *y* are the coordinates of the output pixels, and *k* is the size of the kernel.

1. ReLU (Rectified Linear Unit) Activation Function:

(2) $$ReLU\left(x\right)=max\left(0,x\right)\;$$

2. Max Pooling Layer:

The max pooling operation reduces the spatial dimensions by taking the maximum value from a set of values in the input feature map. Equation (3) represents the functionality of the max pooling, where *P* is the pooled output, *p* is the size of the pooling window, *s* is the stride3, and *I* is the input image.

(3) Px,y=max i=0p−1 max j=0p−1Isx+i,sy+j

3. Bottleneck Block:

The bottleneck block consists of a series of convolutional layers to reduce the number of parameters while maintaining the network’s depth. The bottleneck block with three convolution layers is as follows.

(4) $$Output=Relu\left(Conv\left(1\times 1,\;input\right)\right)$$

(5) $$Output=Relu\left(Conv\left(3\times 3,\;Output\right)\right)$$

(6) $$Output=Relu\left(Conv\left(1\times 1,\;Output\right)\right)$$

The above Equations (4)–(6) represent the functionality of the bottleneck block in the proposed model, where a ReLU activation follows each convolution operation.

4. Global Average Pooling:

The global average pooling reduces each feature map to a single value by taking the average of all the values in the feature map, which is represented by the following equation.

(7) $$GAP=\frac{1}{H\times W}\sum_{i=1}^{H}\;\sum_{J=1}^{W}\;I\left(i,j\right)$$

where *GAP* is the global average pooled output, and *H* and *W* are the height and width of the feature map.

### 3.2. Support Vector Machine (SVM)

This machine learning technique is widely used in the regression and classification of brain tumors. It involves finding a hyperplane to separate different groups based on characteristics. In brain tumor classification, it extracts characteristics from images and categorizes them into groups like glioma, meningioma, pituitary, or no tumor. The goal is to find the best border to separate different classes. The classifier is defined in the terms of the equations below.

(8) $$f\left(x\right)=w^{T}\;x+b$$

In Equation (8) above, *w* is the weight vector, *x* is the input feature vector, and *b* is the bias term.

Class = SVM (Global Avg Pool Output)(9)

The network utilizes convolutional, max pooling, bottlenecking, and global average pooling layers to process the input image. It then classifies the generated feature vector using an SVM classifier into one of the output classes, such as glioma, meningioma, pituitary, or no tumor.

#### Experimental Setup

The experiments were conducted on a Linux server with an NVIDIA RTX 2080 GPU running CUDA 11.3 (NVIDIA, St. Clara, CA, USA) for GPU acceleration and using Python 3.9 with libraries such as TensorFlow 2.5.0 for the MobileNetV2 model, scikit-learn 0.24.2 for the SVM classifier, and NumPy 1.19.5 and Panda 1.2.4 for data processing.

### 3.3. Brain Tumor Classification Methodology

**Figure 2:**
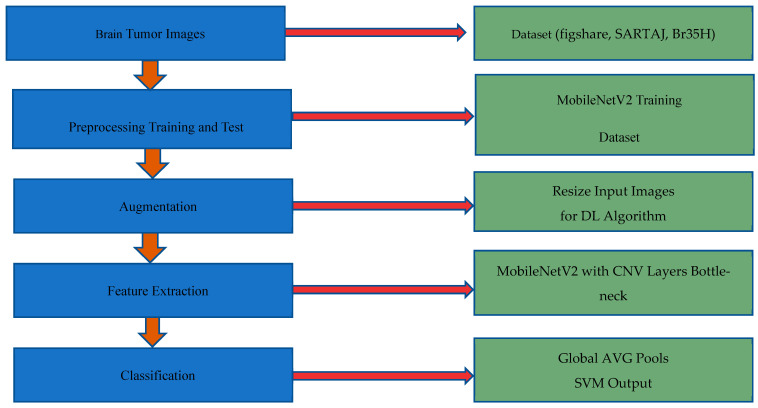
Data flow diagram of the proposed model.

The classification system has many stages: data acquisition, data preprocessing process, dataset augmenting, feature extraction process, classification, and recognition. Figure 2 illustrates the detailed stages of data flow involved in the proposed model.

## 4. Datasets Description

The dataset consists of 7023 MR images obtained from figshare, SARTAJ, and Br35H datasets acquired from Kaggle. The dataset is classified into 80% for training, 20% for testing, and 20% for validation of the training set. The training dataset has four discrete classes: glioma (1321 images), meningioma (1339 images), no tumor (1595 images), and pituitary (1457 images). The testing dataset consists of glioma (300 images), meningioma (306 images), no tumor (405 images), and pituitary (300 images).

The Kaggle MR images dataset, while useful for this study, lacks specific details on patient demographics, tumor stages, and imaging conditions. Greater diversity in these factors could enhance the model’s generalizability, making it more robust across different patient populations and clinical settings. Incorporating more varied datasets in future work would likely improve the model’s applicability.

### 4.1. Distribution of MR Images of Different Brain Class

Table 2 depicts the distribution of MR (Magnetic Resonance) images classified into various groups and the number of images in each group used for training, validation, and testing, respectively. The categories are glioma, meningioma, no tumor, and pituitary.

### 4.2. Datasets Preprocessing and Model Training

**Figure 3:**
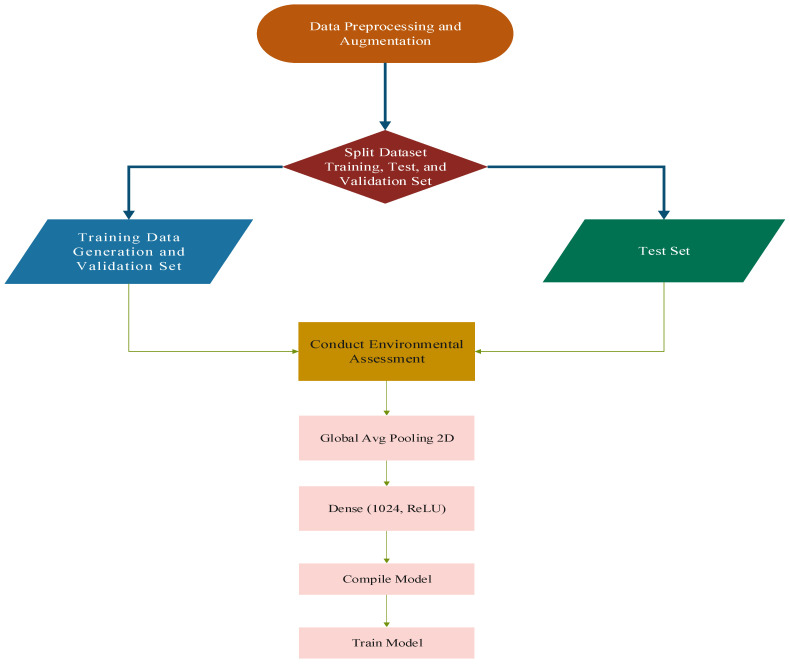
Dataset splitting and training process of the proposed approach.

The dataset preprocessing and model training pipeline for brain tumor classification using MobileNetV2 and an SVM classifier is depicted in Figure 3. Initially, data preprocessing and augmentation are performed to enhance the quality and diversity of the dataset, ensuring robustness against overfitting and improving generalization. The dataset is subsequently divided into three subsets: training, validation, and test sets. Training data undergoes further preparation, including data augmentation and validation, to optimize the input for model training. An environmental assessment step is integrated to ensure that the data preparation aligns with the intended classification task. The model architecture leverages the pre-trained MobileNetV2 backbone, incorporating a global average pooling layer, followed by a dense layer with 1024 neurons and ReLU activation. The compiled model is then trained on the preprocessed dataset, enabling effective feature extraction and classification. This pipeline ensures a systematic approach to brain tumor classification while leveraging state-of-the-art deep learning techniques.

### 4.3. Datasets Preprocessing

The dataset is trained using the MobileNetV2 deep learning algorithm in the data processing phase. The current investigation introduces a reliable technique for classifying brain tumors based on magnetic resonance (MR) imaging. A Support Vector Machine (SVM) classifier enhances its performance. The hybrid model we developed, which integrates deep learning and efficient machine learning, enhances diagnostic processes’ speed and reliability. This feature makes it a crucial instrument in clinical scenarios.

### 4.4. Data Augmentation and Rationale for Model Selection

To enhance model performance and generalization, several data augmentation techniques were applied, including random rotations, horizontal and vertical flips, and zooming. These techniques helped simulate variations in tumor presentation, improving the model’s ability to generalize across different image conditions. MobileNetV2 was selected as the feature extractor due to its efficient architecture, utilizing depth-wise separable convolutions to reduce computational complexity while maintaining strong feature extraction capabilities. This makes it particularly suitable for medical image analysis with large-scale data. For the classification step, Support Vector Machine (SVM) was chosen for its robustness in handling high-dimensional data and its ability to create an optimal hyperplane for separating classes. SVM’s margin-based approach offers better generalization than standard classifiers like SoftMax, particularly in cases with class imbalances, making it ideal for this task. MobileNetV2 and SVM form a hybrid model combining efficient feature extraction with precise classification.

### 4.5. Feature Extraction

Feature extraction must be conducted in order to provide input to the classifiers. This research employs machine learning as well as neural network algorithms. Different feature extraction methodologies are used, namely the pre-trained MobileNetV2 model, pre-trained MobileNetV2 block-wise features, and training the model from the early defrost phase.

### 4.6. Classification

A classifier categorizes data into different classes using a training set and associated values. It can then classify fresh data based on learned patterns. Tumor categorization of brain MRI can be achieved by incorporating brain feature data into machine learning algorithms. The obtained features are trained using machine learning methods like SoftMax, SVM, and Decision Tree. Evaluations compare methods and the results are obtained using a confusion matrix. In multi-class classification tasks, the SoftMax function transforms model outputs into probabilities, representing the probability of an input belonging to a specific output class.

(10) $$P\left(y=j|z\right)=\frac{e^{zj}}{\sum_{k=1}^{k}\;e^{zk}}$$

This represents the probability that a given input *z* (a vector of raw class scores from a model) belongs to class j. Here, $e^{zj}$ is the exponential of the score for class *j* and the denominator is the sum of exponentials of all class scores, which normalizes the output to be a probability distribution across *K* classes. The SVM works by successfully identifying the best hyperplane that can help divide the dataset into different classes.

(11) w×x+b=0

where *w* represents the weight vector, *x* is the feature vector of an input sample, and *b* is the bias.

### 4.7. Dataserts Sample Images

**Figure 4:**
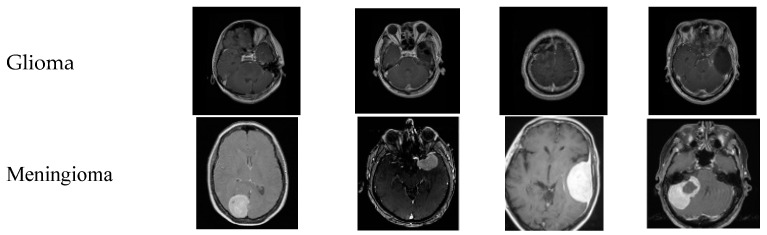

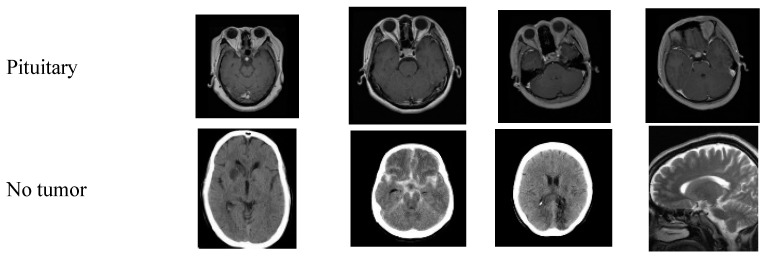
Sample images of the utilized dataset.

Figure 4 shows the sample images of every class; the first row represents the sample MR images of class glioma, the second row has the meningioma class, the third row represents the pituitary class, and the fourth row represents the no tumor class. The images used in the no tumor class were obtained from the Br35H dataset. This dataset offers an extensive and varied collection of images to train and evaluate the models used in this study, enabling a more comprehensive analysis of the effectiveness of the models. Figure 4 displays a small selection of images from the dataset presented.

### 4.8. Hyperparameter Turning

The performance of the proposed hybrid model was significantly influenced by the careful selection and optimization of hyperparameters for both MobileNetV2 and SVM. For MobileNetV2, we used a learning rate of 0.0001 and optimized the model using the Adam optimizer, which is effective for handling large datasets and sparse gradients. The batch size was set to 32, and we applied dropout regularization at a rate of 0.5 to prevent overfitting.

For the SVM classifier, we performed hyperparameter tuning using grid search. Key hyperparameters included the kernel type (with the radial basis function (RBF) and linear kernels tested), the regularization parameter (C), and the gamma parameter (specifically for the RBF kernel). The grid search technique allowed us to systematically explore combinations of these parameters and select the optimal values that maximized classification accuracy.

### 4.9. Handling Data Imbalance

Data imbalance presents a substantial obstacle in the classification of medical images. To tackle this problem, various methodologies were utilized. Data augmentation was employed to artificially augment the quantity of data in underrepresented groups by implementing modifications such as rotation, flipping, and scaling. In addition, the class weights were modified during the model’s training to prioritize the minority classes and give them greater attention. These tactics aid in reducing the effects of data imbalance and improving the overall performance of the model.

### 4.10. Preventing Overfitting

The fusion of MobileNetV2 and SVM led to increased complexity and overfitting risk. To improve resilience, data augmentation and dropout layers were used. Cross-validation was performed to verify the model’s generalization ability to new and unknown data. Regularization techniques, specifically L2 regularization, were applied to the SVM to mitigate overfitting risk, ensuring a balance between model complexity and generalization ability.

## 5. Results

### 5.1. Confusion Metrics

**Figure 5:**
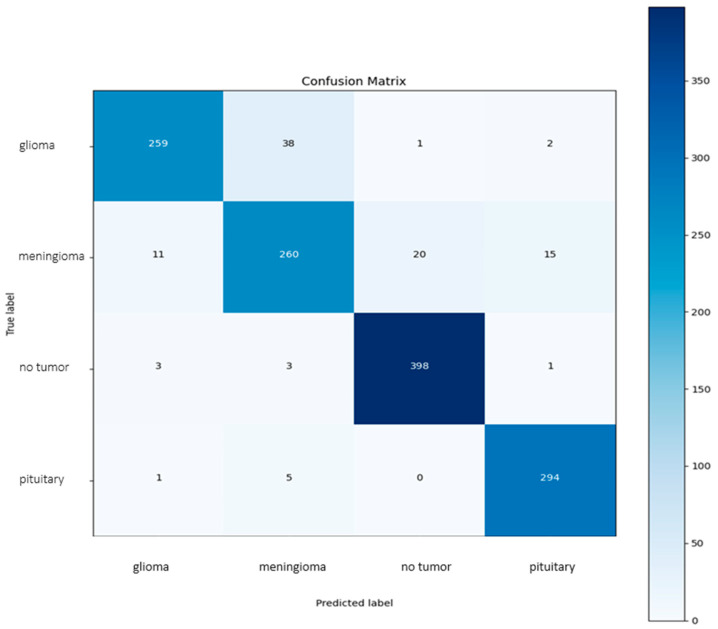
Confusion metrics.

Figure 5 represents the model performance in terms of confusion metrics. The predicted number of images in the test set is obtained as unseen data, and overall, the prediction of the model is better. The confusion matrix below will give a comprehensive analysis of how effective the classification model is in diagnosing diverse forms of brain tumors: glioma, meningioma, and pituitary tumors, along with no tumor cases. The prediction level accuracy of the model is very good, as most of the predictions made by the model correlate to a greater extent with actual labels, especially in cases of meningioma and pituitary tumors.

The true positive count for both these categories is high, suggesting that the model is outstanding in differentiating both forms of tumors. Nonetheless, there do exist some misclassifications that need to be looked into to improve the accuracy of the model. Therefore, at this boundary, most of the errors are expected to occur, showing the areas of overlap of features used to distinguish between the classes of glioma and meningioma. Generally, a very good level of performance is shown by the overall confusion matrix plot.

### 5.2. Performance Metrics of the Proposed Model

The ablation study highlights the importance of combining MobileNetV2 and SVM, demonstrating that the hybrid model outperforms its individual components and prior fusion techniques. Our suggested model exhibits superior precision, F1 score, sensitivity, and specificity indicating its resilience and usefulness in classifying brain tumors.

### 5.3. Proposed Model Analysis

**Figure 6:**
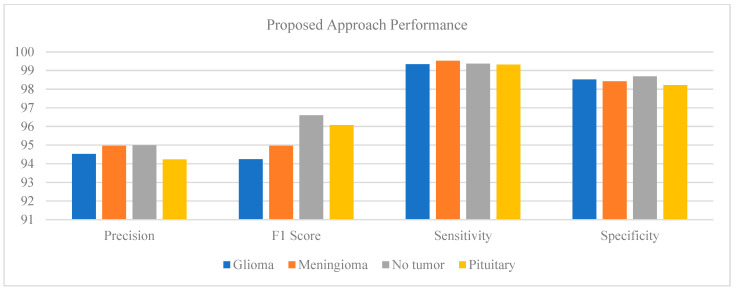
Proposed approach performance.

Figure 6 represent the statistical analysis of the proposed deep learning-based model in terms of precision, F1 score, sensitivity, and specificity. The work uses MobileNetV2 architecture and a Support Vector Machine (SVM) classifier to classify the tumors. There are four classes, glioma, meningioma, no tumor, and pituitary, in the dataset. Each class has its specific imaging markers, which play a crucial role in the proper course of the learning process. In this present work, an evaluation of the proposed model was performed using several metrics designed from a confusion matrix: precision, F1 score, sensitivity (also called recall), and specificity. The model presented good precision for all classes, showing very few false positives.

The precision rate for classifying glioma and identifying pituitary tumors was 94.53% and 94%, respectively. With such specificity of about 97.5% and 100%, respectively, for the above tumors, the model was very accurate in predicting the two types of tumors mentioned. The F1 score, based on precision and sensitivity, gives high performance for the no tumor and pituitary class: 96.60% and 96.08%, respectively.

This shows great promise in identifying tumors correctly and recognizing their absence. The no tumor and pituitary classes have shown the maximum model sensitivity in correctly identifying the actual positive cases, proving the model’s effectiveness in detecting those situations when they are present. Other than that, the specificity ratings were excellent for all classes, with all scores being more than 95%. The greatest specificity of 98.52% was met within the glioma class.

This represents the model’s capability to filter out effectively those cases in which the class is not present, hence decreasing the false alarm rate. This makes a total of the MobileNetV2–SVM model applied with a strong ability to classify brain cancers on MRI data. The combination of such a strong deep learning framework for the extraction of features with a machine learning model for high accuracy in the ability to recognize and categorize complex patterns met within medical imaging leads to the strong possibility of this model to be an excellent tool for aid in clinical diagnosis, if not being definitive, along with a base for further research in medical image analysis.

**Figure 7:**
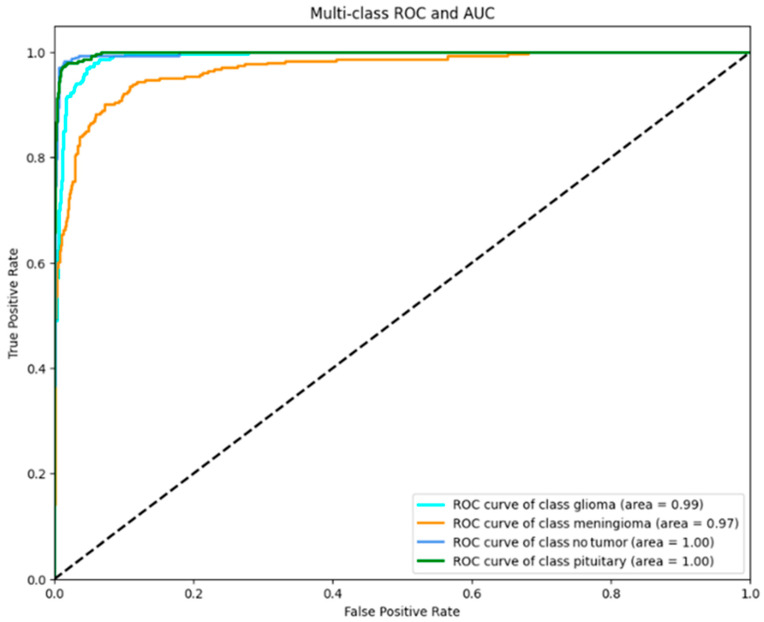
Proposed model ROC representation.

Figure 7 represents the receiver operating characteristic (ROC) curve plot for the MobileNetV2 with SVM classifier, demonstrating outstanding diagnosis accuracy for the four unique classes: glioma, meningioma, no tumor, and pituitary.

Glioma: This class is shown by a curve that converges towards the upper-left corner, exhibiting an AUC (Area Under the Curve) value of 0.99, which suggests an almost flawless level of sensitivity and specificity. Accurate diagnosis is indispensable in the planning of effective treatment against gliomas, a prevalent and severe form of brain tumor. This class performance, therefore, consistently distinguishes instances of glioma against other forms, which is indispensable to the patient’s outcome.

Meningioma: This classifier can recognize a meningioma, a common tumor derived from the meninges, the membrane layers that cover the central nervous system. It is able, with an AUC value of 0.97. Although generally not life-threatening, meningioma presents serious health risks if it is allowed to grow. The high accuracy of the model ensures that most cases are correctly classified as meningioma, hence assisting the implementation of early intervention methods.

No tumor: The blue curve precisely aligns with the left-hand and top limits of the ROC space for the ‘notumor’ class, indicating an AUC equal to 1.00. This is essential to the model, as it ensures that medical procedures and stress are not carried out on healthy persons. The model’s perfect performance in identifying the correct tumor-free scan exhibits great promise as a reliable non-invasive tool.

Pituitary: The class of pituitary tumor ROC curve obtains a perfect AUC of 1.00. Generally, pituitary tumors are benign and mostly do not produce any sign of ailment, though they can cause hormone abnormalities, among other health issues. The ability of the model to assess the presence of pituitary tumors may, in turn, help in the effective and timely control of the disease, hence reducing the attendant effects on a patient’s quality of life.

The strong AUC values of the MobileNetV2 with SVM model in all the classes demonstrate the outstanding reliability of the model to discriminate between different types of tumors and differentiate healthy cases. The top-of-the-line performance of the model puts it at the forefront of modern AI technology in medical imaging and, therefore, represents a potentially powerful primary diagnosis tool that might contribute to cases where acceleration of treatment would be possible, taking the strain off healthcare systems.

The model, MobileNetV2 with SVM, is a great breakthrough in the implementation of artificial intelligence toward correct medical diagnosis. The proposed multi-class model’s structure detects the presence of glioma, meningioma, and pituitary cancers properly and indicates the healthy class of people without tumors, having accuracy and generalizability. The performance of this product is well balanced to enhance the suitability of its incorporation into clinical processes, thus helping medical professionals deliver accurate and customized treatment.

In addition to the high AUC scores reported for each class, other performance metrics such as precision, recall, F1 score, and the confusion matrix provide a more comprehensive evaluation of the model. As shown in Table 3, the precision and recall for glioma were 0.90, meningioma was 0.84, and pituitary was 0.96, and no tumor was 0.96, respectively, with an F1 score of 99%. These metrics indicate the model’s strong performance in correctly identifying true positives while minimizing false positives, especially for critical tumor categories such as glioma and pituitary tumors.

Generally, this model’s high precision level, as indicated by AUC and ROC, is in all classes. This could mean that it is a reliable tool that can favorably affect patient treatment routes.

#### Discussion

In this research, the MobileNetV2 pre-trained deep learning model for feature extraction and SVM for classification are trained on a fused dataset (figshare, SARTAJ, Br35H). The model’s performance is assessed by classifying brain tumors in the dataset as gliomas, meningiomas, pituitary tumors, and tumor tumors. According to the experiment’s results, the performance of the MobileNetV2 transfer model is robust.

The assessment phase should thoroughly analyze the performance of the combined MobileNetV2–SVM model using the provided confusion matrix. The primary objective of this evaluation is to assess the model’s ability to classify various brain tumor types present in the dataset accurately. By examining the true positive rates in the confusion matrix, it is evident that the MobileNetV2 model effectively recognizes the unique characteristics of each tumor category. Furthermore, the SVM classifier distinguishes characteristics between all classes with high confidence.

The proposed MobileNetV2–SVM hybrid model not only demonstrates high classification accuracy but also holds significant potential for improving clinical practices in diagnosing brain tumors. By automating the classification of MRI images, the model can assist radiologists and clinicians in making quicker and more accurate diagnoses, particularly for early-stage tumors that may be more difficult to detect manually. The model’s high AUC scores across multiple tumor types suggest that it can effectively differentiate between benign and malignant tumors, reducing the risk of misdiagnosis and unnecessary interventions.

Additionally, MobileNetV2’s lightweight architecture makes the model feasible for deployment in resource-constrained environments, such as smaller clinics or mobile diagnostic tools. This could extend advanced diagnostic capabilities to regions with limited access to expert radiologists or high-end medical equipment. Integrating this model into clinical workflows could lead to more standardized and efficient diagnosis processes, ultimately improving patient outcomes by enabling earlier detection and treatment of brain tumors.

### 5.4. Different Pre-Trained Models with the Proposed Model

As shown in Table 4, the proposed MobileNetV2–SVM model exhibits superior performance in terms of specificity and F1 score for glioma and pituitary tumors when compared to models such as BT3_VGG16 and BT3_ResNet101. This highlights the model’s ability to effectively reduce false positives while maintaining a balanced precision–recall tradeoff, which is particularly critical in medical applications where diagnostic accuracy is essential.

From the assessment results displayed in Table 4, it has been represented that performance measures clearly have different values for categorizing different models under some models. Among these models, the proposed MobileNetV2 + SVM algorithm is the most remarkable one due to the key improvements it brings and the novel approach it adopts. In this proposed method, a very efficient MobileNetV2 architecture is used for feature extraction, which is further combined with a very strong classification power of the Support Vector Machine (SVM) classifier; these results are outstanding on all evaluation metrics. It is also possible that there may be higher sensitivity, specificity, accuracy, and F1 scores for the output of the models as compared to prior ones, particularly for the identification of glioma and pituitary tumor types. Further, the collaboration of MobileNetV2 with SVM ensures improved classification accuracy but also promises effectiveness and robustness; thus, it is a very good candidate for use in tumor classification tasks.

This proposed approach consolidates the advantages of MobileNetV2 and SVM and hence exhibits a powerful combination of efficiency, accuracy, and generalization capability. This offers the potential for remarkable improvement in the specific domain of medical image analysis for tumor detection and its classification.

So, the proposed method of MobileNetV2 + SVM performs comparably well against other methods of evaluation, showing excellence in most of the metrics, such as sensitivity, specificity, precision, and F1 score. The model depicts a high-performance level in the detection and discrimination of glioma, pituitary, and no tumor classes regarding sensitivity, specificity, accuracy, and F1 score compared to other models. This is a demonstration of the effectiveness of the algorithm in the accurate diagnosis of certain types of tumors from medical imaging.

The approach relies firmly on the use of MobileNetV2, which has proved itself to be very efficient and effective in the task of image classification. The architecture of the system considers depth-wise separable convolutions and linear bottleneck structures, which are low in terms of computing cost and are performance-preserving. On a broad spectrum, this architecture falls into very efficient structures for the purpose of extracting features from medical images within the tumor classification domain. It is able to capture spatial patterns and structures that are indicative of various types of tumors. Afterward, through this segmentation, features are extracted from the input image and further classified. As described using features extracted by MobileNetV2, it is able to produce distinctions across tumor classifications away from high accuracy and completeness. This combination of feature extraction with classification improves the generalization capability of the algorithm since it classifies the tumors specifically in regard to their specific features.

### 5.5. Proposed Model Comparison with Pretrained Models

**Figure 8:**
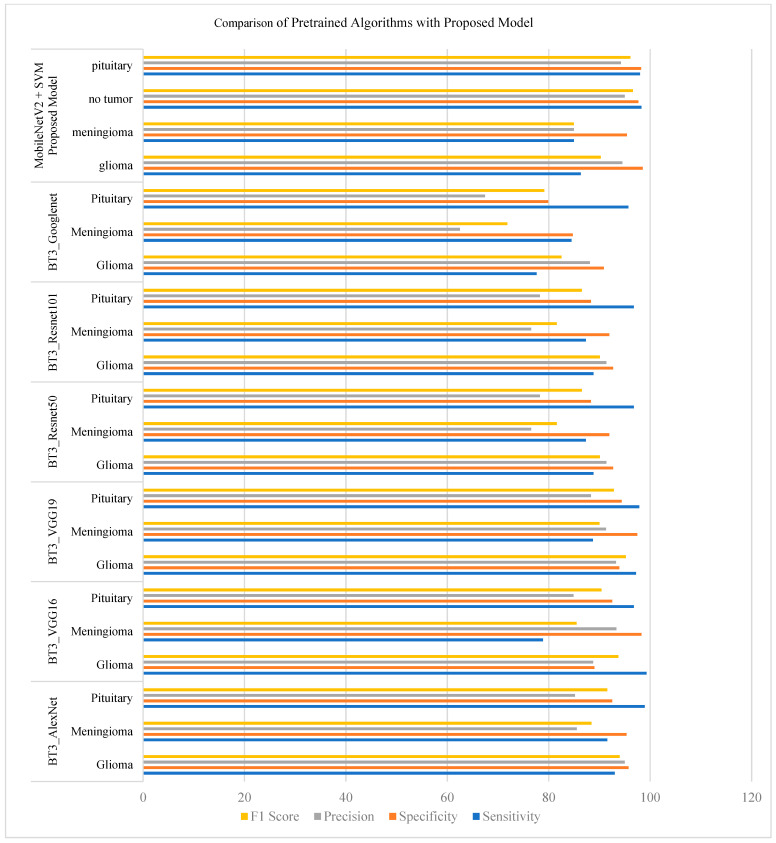
Comparison of pretrained algorithms with proposed model.

Figure 8 shows the analysis comparing the previously employed deep learning methods with the proposed model. In this analysis, the proposed model presents outstanding results regarding precision and F1 score for the pituitary and tumor classes.

### 5.6. Computational Cost and Real-Time Applications

Integrating SVM with MobileNetV2 improves classification accuracy but can increase computing workload. To address this, MobileNetV2’s lightweight architecture, utilizing depth-wise separable convolutions, significantly reduces computational load, making it suitable for resource-constrained environments. Although SVM may be computationally expensive, using pre-extracted features from MobileNetV2 minimizes the dimensionality of the input, reducing the classification cost. In our experiments, the model processed MRI images efficiently on an NVIDIA RTX 2080 GPU, demonstrating feasibility for real-time applications. To further optimize SVM and reduce the workload, lightweight alternatives and hardware accelerations like GPUs could be explored alongside the implementation of efficient coding standards. This ensures the model’s balance between accuracy and efficiency, making it deployable in environments with limited computational resources, such as mobile or edge devices.

### 5.7. Limitations and Future Work

While the proposed MobileNetV2–SVM hybrid model demonstrates strong performance on the Kaggle MRI brain tumor dataset, further validation is necessary to ensure its generalizability across different real-world data distributions. To this end, future work will focus on testing the model on additional widely recognized datasets, such as BraTS and TCIA, which contain more diverse and complex brain tumor images. Validating the model on these datasets will provide greater insights into its robustness and adaptability in clinical settings. This additional testing will help confirm the model’s effectiveness in identifying various tumor types across multiple imaging protocols and institutions.

## 6. Conclusions

In this study, we developed a robust MobileNetV2–SVM hybrid model for the classification of brain tumors using MRI images. The model demonstrated superior accuracy, with AUC scores of 0.99 for glioma, 0.97 for meningioma, and 1.0 for pituitary tumors and no tumor categories. These results indicate that our approach outperforms many conventional machine learning methods, offering a highly efficient and accurate solution for tumor classification. Our research highlights the potential of combining deep learning architectures, such as MobileNetV2, with traditional machine learning classifiers like SVM to improve diagnostic outcomes. By streamlining the tumor classification process, this method can facilitate more accurate early diagnoses, reduce the likelihood of misdiagnosis, and ultimately enhance patient outcomes.

The findings from this study also overlay the way for further exploration in this area. Future work could expand on our model by testing it on more diverse datasets, incorporating additional deep learning techniques, and optimizing its performance for real-time clinical applications. This research contributes to the ongoing development of medical imaging technologies and sets the stage for more efficient and reliable diagnostic tools in healthcare.

## Figures and Tables

**Table 1:** Summary of the existing literature.

Study	Techniques Used	Advantages	Limitations
Combined Channel and Spatial Attention-Based Stereo Endoscopic Image Super-Resolution	Channel and Spatial Attention Mechanisms	Enhanced Image Quality	Computationally Intensive
Saliency-Aware Deep Learning Approach for Enhanced Endoscopic Image Super-Resolution	Saliency Detection, Deep Learning	Improved Resolution	Requires Large Training Data
Automatic segmentation of large-scale CT image datasets	CT Image Segmentation	Detailed Analysis	Data Intensive

**Table 2:** Distribution of MR images across different classes.

Class	Number of Images		
	Training Data	Validation Data	Testing Data
Glioma	1056	265	300
Meningioma	1071	268	306
No tumor	1276	319	405
Pituitary	1165	292	300

**Table 3:** Performance metrics of the proposed model.

Class	Precision	F1 Score	Sensitivity	Specificity
Glioma	0.9453	0.9024	0.8633	0.9852
Meningioma	0.8497	0.8497	0.8497	0.9542
No tumor	0.9499	0.9660	0.9827	0.9768
Pituitary	0.9423	0.9608	0.9800	0.9822

**Table 4:** Comparison of the baseline with the proposed model.

Baseline	Tumor Type	Sensitivity	Specificity	Precision	F1 Score
BT3_AlexNet	Glioma	93.00	95.73	95.00	93.99
	Meningioma	91.54	95.33	85.52	88.43
	Pituitary	98.92	92.52	85.18	91.54
BT3_VGG16	Glioma	99.30	89.02	88.75	93.72
	Meningioma	78.87	98.30	93.33	85.49
	Pituitary	96.77	92.52	84.90	90.40
BT3_VGG19	Glioma	97.20	93.90	93.28	95.20
	Meningioma	88.73	97.45	91.30	90.00
	Pituitary	97.84	94.39	88.34	92.85
BT3_Resnet50	Glioma	88.81	92.68	91.36	90.07
	Meningioma	87.32	91.94	76.54	81.57
	Pituitary	96.77	88.31	78.26	86.53
BT3_Resnet101	Glioma	88.81	92.68	91.36	90.07
	Meningioma	87.32	91.94	76.54	81.57
	Pituitary	96.77	88.31	78.26	86.53
BT3_Googlenet	Glioma	77.62	90.85	88.09	82.52
	Meningioma	84.50	84.74	62.50	71.85
	Pituitary	95.69	79.90	67.42	79.11
Proposed Model MobileNetV2 + SVM	Glioma	86.33	98.52	94.53	90.24
	Meningioma	84.97	95.42	84.97	84.97
	No Tumor	98.27	97.68	94.99	96.60
	Pituitary	98.00	98.22	94.23	96.08

## Data Availability

The original contributions presented in the study are included in the article, further inquiries can be directed to the corresponding author.
